# Newly Synthesized CoFe_2−y_Pr_y_O_4_ (y = 0; 0.01; 0.03; 0.05; 0.1; 0.15; 0.2) Nanoparticles Reveal Promising Selective Anticancer Activity Against Melanoma (A375), Breast Cancer (MCF-7), and Colon Cancer (HT-29) Cells

**DOI:** 10.3390/nano15110829

**Published:** 2025-05-30

**Authors:** Slaviţa Rotunjanu, Roxana Racoviceanu, Armand Gogulescu, Alexandra Mioc, Andreea Milan, Narcisa Laura Marangoci, Andrei-Ioan Dascălu, Marius Mioc, Roxana Negrea-Ghiulai, Cristina Trandafirescu, Codruţa Șoica

**Affiliations:** 1 Department of Pharmacology-Pharmacotherapy, Faculty of Pharmacy, Victor Babes University of Medicine and Pharmacy, Eftimie Murgu Square, No. 2, 300041 Timișoara, Romania; slavita.rotunjanu@umft.ro (S.R.); alexandra.mioc@umft.ro (A.M.); roxana.ghiulai@umft.ro (R.N.-G.); codrutasoica@umft.ro (C.Ș.); 2 Research Center for Experimental Pharmacology and Drug Design (X-Pharm Design), Victor Babes University of Medicine and Pharmacy, Eftimie Murgu Square, No. 2, 300041 Timișoara, Romania; babuta.roxana@umft.ro (R.R.); andreea.milan@umft.ro (A.M.); marius.mioc@umft.ro (M.M.); 3 Department of Pharmaceutical Chemistry, Faculty of Pharmacy, Victor Babes University of Medicine and Pharmacy, Eftimie Murgu Square, No. 2, 300041 Timișoara, Romania; trandafirescu.cristina@umft.ro; 4 Department XVI, Balneology, Medical Rehabilitation and Rheumatology, Victor Babes University of Medicine and Pharmacy, 2 Eftimie Murgu, 300041 Timisoara, Romania; 5 Institute of Macromolecular Chemistry Petru Poni, 700487 Iasi, Romania; nmarangoci@icmpp.ro (N.L.M.); idascalu@icmpp.ro (A.-I.D.)

**Keywords:** praseodymium-doped magnetic nanoparticles, cobalt ferrites, cytotoxicity, cell viability, anticancer

## Abstract

In this study, praseodymium-doped cobalt ferrite nanoparticles (CoFe_2−y_Pr_y_O_4_, y = 0–0.2) were synthesized via sol-gel auto-combustion and systematically characterized to assess their structural, morphological, magnetic, and biological properties. X-ray diffraction (XRD) confirmed single-phase cubic cobalt ferrite formation for samples with y ≤ 0.05 and the emergence of a secondary orthorhombic PrFeO_3_ phase at higher dopant concentrations. FTIR spectroscopy identified characteristic metal–oxygen vibrations and revealed a progressive shift of absorption bands with increasing praseodymium (Pr) content. Vibrating sample magnetometry (VSM) demonstrated a gradual decline in saturation (Ms) and remanent (Mr) magnetization with Pr doping, an effect further intensified by cyclodextrin surface coating. TEM analyses revealed a particle size increase correlated with dopant level, while SEM images displayed a porous morphology typical of combustion-synthesized ferrites. In vitro cell viability assays showed minimal toxicity in normal human keratinocytes (HaCaT), while significant antiproliferative activity was observed against human cancer cell lines A375 (melanoma), MCF-7 (breast adenocarcinoma), and HT-29 (colorectal adenocarcinoma), particularly in Pr 6-CD and Pr 7-CD samples. These findings suggest that Pr substitution and cyclodextrin coating can effectively modulate the physicochemical and anticancer properties of cobalt ferrite nanoparticles, making them promising candidates for future biomedical applications.

## 1. Introduction

Introduced as a concept in 1959, nanotechnology is now considered the most promising technology of the 21st century, including in the medical field where nanoparticles may serve as novel diagnostic tools, targeted drug carriers, or biomedical implants [1]. Among various types of nanoparticles, magnetic nanoparticles display unique properties such as diverse physicochemical parameters, easy synthesis, biocompatibility, and stability that render them suitable for biomedical purposes [2]. Magnetic nanoparticles are formed from metal elements or their oxides; superparamagnetic magnetite (Fe_3_O_4_) is the most commonly used, given its high biocompatibility [2,3]. Partial substitution of iron with other metals, such as Co, Ni, K, etc., produces ferrites that can be classified according to their crystal structure and magnetic properties; spinel ferrites display the general formula M^2+^Fe_2_^3+^O_4_ and have unique multifunctional properties such as strong magnetic behavior, high specific surface area, active sites at the surface allowing for further functionalization, and chemical stability, as well as various shapes and sizes [4]. Unlike spinel ferrites, where the divalent metal ion occupies tetrahedral sites while the iron ion Fe^3+^ can be found in octahedral sites, in cobalt ferrites (CoO·Fe_2_O_3_), the Co^2+^ ions are distributed in octahedral sites, with the iron ions equally distributed in tetrahedral and octahedral sites, which produces an inverse spinel structure [5] whose properties can be altered through the composition and synthesis method.

Cobalt ferrite nanoparticles have been intensively studied due to their specific chemical, physical, electrical, and mechanical properties that provide them with unique features, which allow for their application in various fields [6]. Unlike iron oxide nanoparticles that are prone to aggregation and oxidation and display renal and liver toxicity through oxidative cell damage, cobalt ferrite nanoparticles lack such toxic effects even at high doses necessary in hyperthermia [7]. Although frequently assessed as drug carriers, cobalt ferrites can act as anticancer agents themselves, presumably due to their cytotoxic effects that occur after cellular uptake due to a pH-dependent release of Co^2+^ ions [8]. Moreover, when comparing Co^2+^ ions generated by cobalt chloride and cobalt ferrite, respectively, the study showed that intrinsic cobalt toxicity prevents its use as a monotherapy antitumor agent.

Cobalt ferrite nanoparticles were identified as antiproliferative agents against MCF7 breast cancer cells while lacking cytotoxic effects in HEK-293 human embryonic kidney cells, thus displaying selective anticancer activity [9]. Similar results were reported in HCT-116 human colorectal cancer cells [10]. Studies show that at low concentrations, the nanoparticles accumulate in the perinuclear region within cells; at increased concentrations, Co and Fe are present in the nuclear region, triggering cell morphological alterations, presumably due to an increased Co/Fe ratio as a result of Co biodegradation and accumulation in the cell nucleus [11]. However, more studies have been conducted on doped cobalt ferrites with different metal elements in an effort to enhance their properties and, therefore, their biomedical applications. Doping with transitional metals leads to changes in the physical properties, depending on the distribution of doping ions between the two interstitial sites of the cobalt ferrite spinel structure, as well as on their valence [12]. Similarly, doping with large-size rare earth metals produces significant adjustments in the physicochemical features of cobalt ferrites, such as specific surface area and particle size, depending on the type and concentration of the dopant, synthesis method, and cation distribution between the tetrahedral and octahedral sites [13]. Even more importantly, doping may increase the cytotoxicity of cobalt ferrite nanoparticles in cancer cells; substitution of Fe^2+^ with Mn^2+^ decreased MCF7 breast cancer cell viability, presumably due to oxidative stress [14].

Rare earth elements or lanthanides exhibit biological properties similar to those of calcium ions; involved in biomedical studies, they may be used as salts, coordination complexes, radioisotopes, or oxides in cancer imaging and therapy [15]. Rare earth metal-substituted cobalt ferrites can be easily synthesized and display a pure spinel phase and uniform and narrow particle size distribution; the substitution is able to decrease particle size and coercivity [16]. The molecular mechanism underlying the anticancer activity of such nanoparticles involves pore formation within the cell membrane, with strong selectivity versus healthy cells being reported; unlike healthy cells, cancer cells exhibits membrane depolarization that enables electroporation, thus allowing for delivery via induced pores [17]. Pr-doped nanorods decorated with poly-β-cyclodextrin were synthesized as nanocarriers for the delivery of 5-fluorouracil; they displayed high loading efficacy and a strong and selective anticancer activity in MCF7 breast cancer cells [18].

In addition to their promising biological effects, the synthesis of doped cobalt ferrite nanoparticles via methods like sol-gel offers good reproducibility and scalability, which are essential for clinical translation. The ability to synthesize these nanoparticles at low processing temperatures in a controlled, cost-effective, and large-scale manner supports their potential use in various biomedical applications, including cancer therapy [19,20].

The aim of the current study was the synthesis of cobalt ferrite nanoparticles doped with Pr and their assessment as anticancer agents; the nanoparticles were physiocochemically analyzed and tested thereafter on normal HaCaT cells and on A375, MCF-7, and HT-29 cancer cells. The biological assessment revealed that all compounds displayed a strong, dose-dependent cytotoxic activity.

## 2. Materials and Methods

### 2.1. Chemicals

The raw materials used for synthetic purposes were iron nitrate nanohydrate (Fe(NO_3_)_3_·9H_2_O, Merck, Darmstadt, Germany), cobalt nitrate hexahydrate (Co(NO_3_)_2_·6H_2_O, Sigma-Aldrich, Darmstadt, Germany), dysprosium chloride x hydrate (PrCl_3_·xH_2_O, Sigma-Aldrich, Darmstadt, Germany), glycine (C_2_H_5_NO_2_, Merck, Darmstadt, Germany), (2-Hydroxypropyl)-γ-cyclodextrin (Sigma-Aldrich, Darmstadt, Germany), and absolute ethanol (Merck, Darmstadt, Germany).

### 2.2. Synthesis by Combustion Method

In order to synthesize 0.02 moles of cobalt ferrite, 0.04 moles of iron nitrate, 0.02 moles of cobalt nitrate, and 0.09 mole of glycine were used; glycine acted as fuel while the nitrates functioned as oxidizing agents. The salts and the glycine were heated together at 60 °C until the formation of a brown solution. Meanwhile, a porcelain capsule was preheated at around 350 °C using a heating mantle. The mixture was then carefully poured into the capsule, and the heating mantle was kept running at 350 °C. After the entire water evaporated, the mixture became viscous and at some point, self-ignited. The combustion front propagated very fast in the entire mass, with the reaction completing in around 9 s. During the reaction, yellow flames, indicating high temperature, were observed; a black and crumbly powder formed as a result.

The Pr-doped cobalt ferrite was synthesized following the same procedure, with an extra step when Pr chloride was added to the mixture. The amount of cobalt nitrate (0.02 moles) and glycine (0.09 moles) remained unchanged while the Pr and iron nitrate quantities were varied. The Pr and Fe salts used are presented in Table 1. Regarding the reaction development, it is worth noting that white gases were released, the duration increased by 3–4 s, and the final powder exhibited a more voluminous, spongy texture compared to the undoped sample.

**Figure 1:**
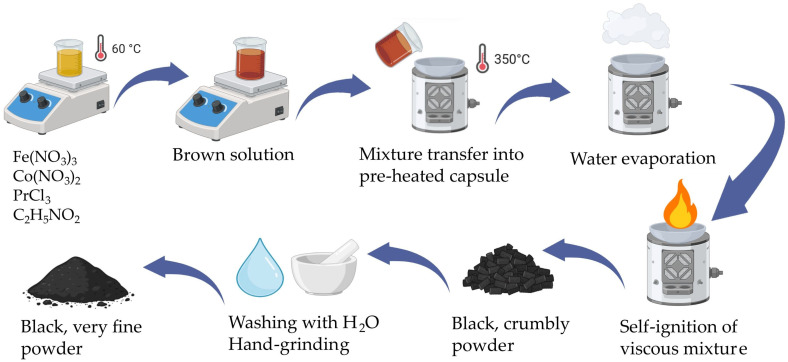
A schematic illustration of the synthesis process of Pr-doped cobalt ferrite nanoparticles (CoFe_2−y_Pr_y_O_4_, y = 0–0.2) via the sol-gel auto-combustion method.

The powders of both doped and undoped cobalt ferrite were washed several times using warm water, then dried and manually ground in a mortar until a very fine powder was obtained (Figure 1).

### 2.3. Cyclodextrin Inclusion Complex

In order to enable biological evaluation, all samples were incorporated into (2-hydroxypropyl)-γ-cyclodextrin, which has the ability to increase their water solubility; a molar ratio of 1:1 cyclodextrin:cobalt ferrite was used. Homogenization was accomplished by using a solvent composed of 0.9 mL distilled water and 2.1 mL absolute ethanol. Further, the mixture was stirred for 30 min and then dried at 70 °C until full solvent evaporation. The resulting complex was then manually ground into a fine powder that was later suspended in distilled water using a UP200S ultrasonic homogenizer (Hielscher Ultrasonics GmbH, Teltow, Germany) for 2 h at 50% amplitude and 0.8 cycles. After sonication, the particles remained suspended without the occurrence of sedimentation.

### 2.4. Characterization Methods

X-ray diffraction (XRD) analysis was performed with a Rigaku SmartLab diffractometer (Tokyo, Japan) equipped with a Cu anode (X-ray wavelength of 1.5406 Å) operating at 45 kV and 200 mA. The sample phase composition was identified using the Crystallography Open Database. Crystallite size was calculated using the Scherrer equation for the (311) peak. NIST 660c LaB6 standard [21] was used for the correction of the instrument-induced broadening. XRD patterns were refined using Rietveld analysis package in the Rigaku SmartLab Studio II software. A Shimadzu IR Affinity-1S spectrophotometer (Shimadzu Scientific Instruments Inc., Columbia, MD, USA) with a 400–4000 cm^−0^ range, 4 cm^−e^ resolution, KBr pellets method, and 40 scans per sample, was used for recording the FTIR spectra. The magnetic properties were evaluated at room temperature and in a magnetic field between 30 and −30 KOe, using a LakeShore 8607 vibrating sample magnetometer (VSM, Shore Cryotronics, Westerville, OH, USA); samples were previously demagnetized in an alternating field. Scanning electron microscopy (SEM) was measured using a Verios G4 UC Scanning Electron Microscope (Thermo Scientific, Brno, Czech Republic). Prior to the measurement, in order to increase electrical conductivity and reduce charge buildup, the samples were coated with 6 nm platinum using a Leica EM ACE200 Sputter coater (Leica Microsystems GmbH, Wetzlar, Germany). SEM investigations were performed using a high-resolution image detector (Through Lens Detector, TLD) at 10 kV, in high vacuum mode. The morphological evaluation was conducted at 120 kV acceleration voltage (Hitachi High-Tech HT7700, Hitachi High-Technologies Corporation, Tokyo, Japan). The instrument was equipped with a STEM module, an energy dispersive X-ray (EDX) detector that allowed for elemental analysis and selected area electron diffraction (SAED) apertures. The samples were prepared by drop casting from their water suspension on 300 mesh carbon-coated copper grids (Ted Pella Inc., Redding, California) and vacuum-dried at room temperature for 24 h.

### 2.5. Cell Culture

Immortalized human keratinocytes HaCaT (CLS Cell Lines Service GmbH, Eppelheim, Germany), human melanoma cells A375, human breast adenocarcinoma cells MCF-7, and human colorectal adenocarcinoma HT-29 cells (American Type Culture Collection ATTC, Lomianki, Poland) were used in the current study. HaCaT and A375 cells were cultured on high-glucose Dulbecco’s Modified Eagle Medium (DMEM) supplemented with 10% fetal bovine serum (FBS) and 1% mixture of penicillin/streptomycin (100 U/mL). McCoy’s 5A supplemented with 10% FBS and 1% antibiotic mixture was employed for culturing the HT-29 cell line, while MCF-7 cells were cultured in Eagle’s Minimum Essential Medium (EMEM) containing the same concentrations of FBS, antibiotic mixture, and 0.01 mg/mL human recombinant insulin. All cells were incubated at 37 °C and 5% CO_2_.

### 2.6. Cell Viability Assessment

The Alamar blue assay was employed to determine the cell viability percentages of HaCaT, A375, MCF-7, and HT-29 cells after stimulation with increasing concentrations (0.025, 0.05, 0.1, 0.25, and 0.5 mg/mL) of the newly synthesized compounds for 48 h. The cells (1 × 10^4^) were seeded onto 96-well plates and incubated up to 85–90% confluence. The medium was removed using an aspiration station and then replaced with fresh medium containing the tested concentrations of each compound. After 48 h, 20 μL Alamar blue 0.01% was added to each well, and the cells were further incubated for another 3 h. To determine cell population, the absorbance of the wells was determined at two wavelengths, 570 nm and 600 nm, using a microplate reader (xMarkTM Microplate, Bio-Rad Laboratories, Hercules, CA, USA). All experiments were performed in triplicate.

### 2.7. Statistical Analysis

To assess statistical significance, we used the one-way ANOVA followed by Dunnett’s post hoc test (GraphPad Prism version 6.0.0, GraphPad Software, San Diego, CA, USA; the differences between the groups were considered statistically significant if *p* < 0.05, as follows: * *p* < 0.05, ** *p* < 0.01, and *** *p* < 0.001.

## 3. Results

### 3.1. X-Ray Diffraction (XRD)

**Figure 2:**
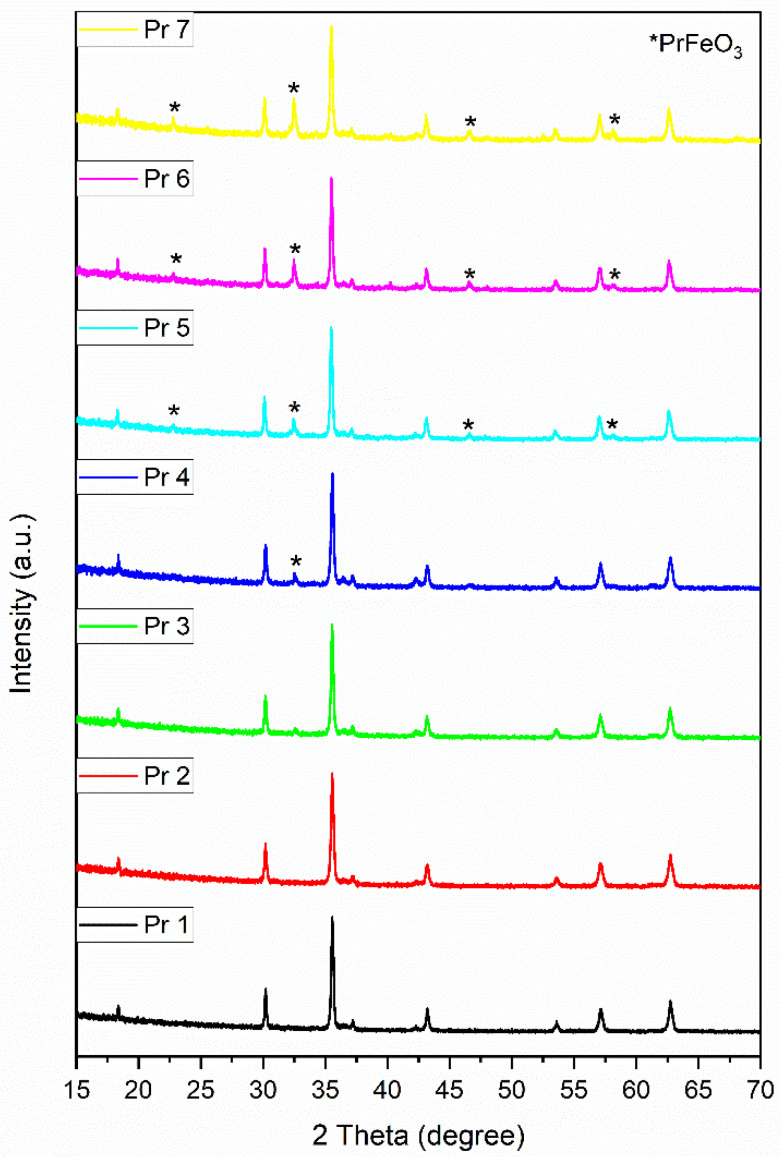
XRD patterns of the samples.

All powder diffraction patterns are well defined, exhibiting intense and narrow diffraction peaks that confirm the high crystallinity of the samples (Figure 2). The undoped sample Pr 1 shows specific diffraction peaks that characterize single-phase cobalt ferrite. The identification of the single-phase was made with the COD card 96-153-3164. The identified diffraction peaks at the 2θ angles 18.32°, 30.14°, 35.51°, 37.13°, 43.15°, 53.55°, 57.09°, and 62.7° were assigned to the crystallographic planes (111), (220), (311), (222), (400), (422), (511), and (440), of cubic cobalt ferrite. No additional phase was observed in the diffractograms of doped samples Pr 2 and Pr 3, which confirms the successful incorporation of Pr in the cobalt ferrite lattice and the definite formation of Pr-substituted cobalt ferrite.

For doped samples Pr 4, 5, 6, and 7, additional diffraction peaks were observed, which indicate the occurrence of a secondary phase identified as perovskite-type crystalline PrFeO_3_ (COD card 96-210-7085). The diffraction peaks located at 2θ angles of 22.78°, 32.55°, 46.61°, and 58.14° correspond to the crystallographic planes (110), (112), (004), and (204) in orthorhombic PrFeO_3_. The intensity of the diffraction peaks assigned to the secondary phase PrFeO_3_ is directly correlated with the initial reagent ratio used in the synthesis reaction. In sample Pr 4, only trace amounts are detected, while in samples Pr 5, Pr 6, and Pr 7, an increased intensity is noticed for the diffraction peaks assigned to the secondary phase.

The lattice parameter *a* for each sample obtained from the Rietveld refinement is presented in Table 1. Starting with the first doped sample Pr 2, the lattice parameter increases as a consequence of the inclusion of the dopant in the cubic network. Furthermore, a gradual increase can be observed for each sample with the increase in Pr^3+^ content. The calculated Scherrer crystallite size evidenced the crystalline character of the samples. The crystallite dimension was influenced by the inclusion of Pr^3+^ into the cubic network; the size was in the narrow range of 43–66 nm (Table 2).

### 3.2. Fourier-Transform Infrared Spectroscopy (FTIR)

**Figure 3:**
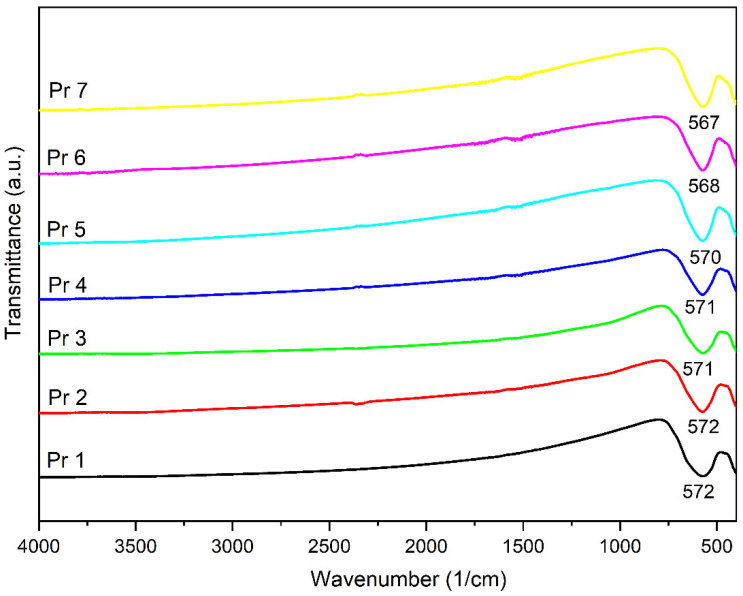
FTIR spectra of the samples.

The FTIR spectra for all samples are displayed in Figure 3; one can notice that all samples show an intense absorption band and a smaller one within the 400–800 cm^−1^ domain, characteristic for the metal–oxygen vibration; also, a small shift was noticed for the absorption maximum due to the Pr presence in the sample.

### 3.3. Investigation of Magnetic Properties (VSM)

**Figure 4:**
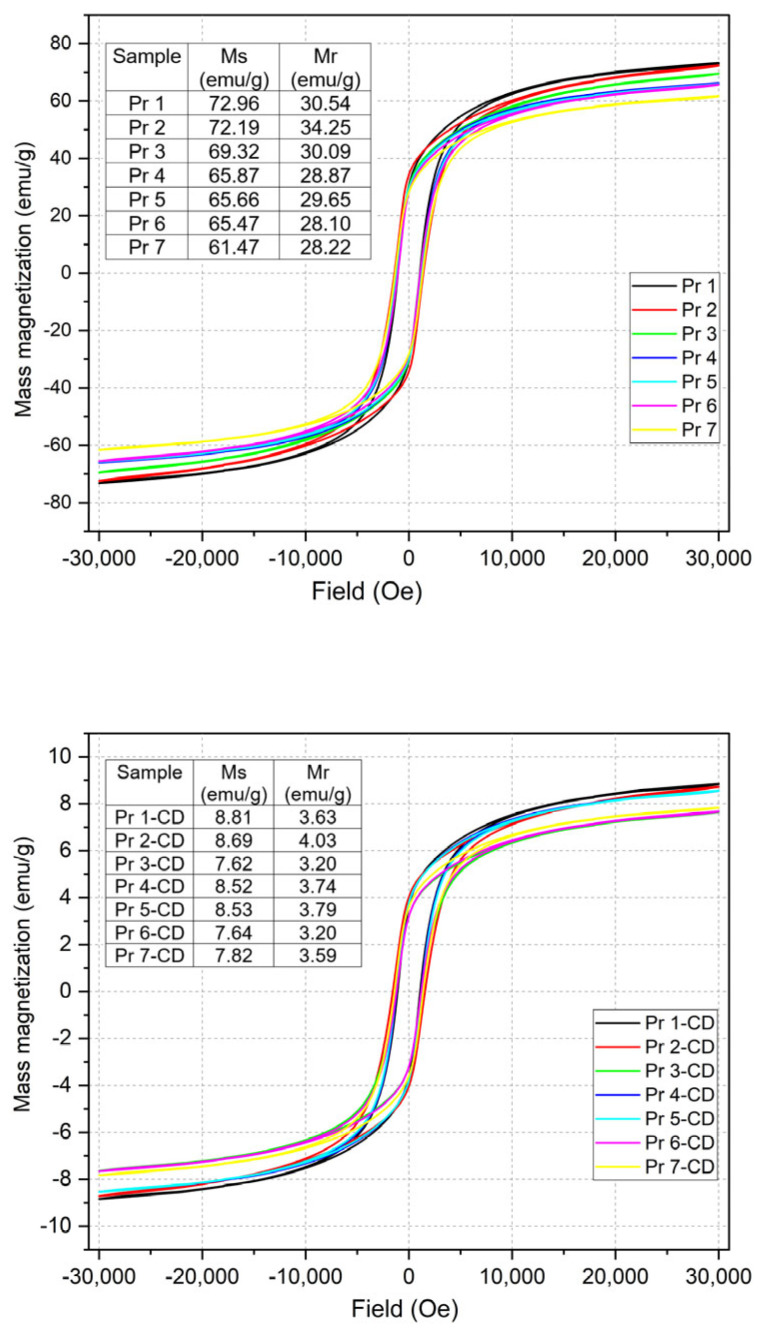
Magnetic hysteresis of the samples.

The magnetic properties of the samples were analyzed, and the hysteresis is presented in Figure 4.

One can notice that all samples display a similar behavior as indicated by resembling characteristics of the hysteresis, with variations in the recorded magnitude. The first two samples have a comparable saturation mass magnetization (Ms) value of approximately 72 emu/g, which decreases with the increase in the Pr content, reaching 61.47 emu/g in sample Pr 7. In terms of remnant mass magnetization (Mr), samples Pr 1 and Pr 3 exhibit a value of 30 emu/g that decreased toward sample Pr 7 (28 emu/g), in a similar trend with the previously reported Ms. Interestingly enough, in samples Pr 2 and Pr 5, out-of-line Mr values of 34 and 29 emu/g, respectively, were recorded.

In cyclodextrin-coated samples, the values of Ms and Mr suffer a significant decrease by comparison to the naked samples; as such, in Pr 1-CD (cobalt ferrite-CD), the respective values dropped to 8.8 emu/g (Ms) and 3.6 emu/g (Mr). In naked samples, the decrease in both Ms and Mr follows a linear trend while in cyclodextrin-coated samples, the same parameters decrease in a more irregular manner; however, in both cases, the strong reduction of Ms/Mr values with increasing amounts of Pr is maintained, with Ms values ranging between 8.8 and 7.6 emu/g and Mr between 4 and 3.2 emu/g. Of note, the presence of cyclodextrin greatly influences the magnetic properties of the sample.

### 3.4. Scanning Electron Microscopy (SEM) and Transmitted Electron Microscopy (TEM)

**Figure 5:**
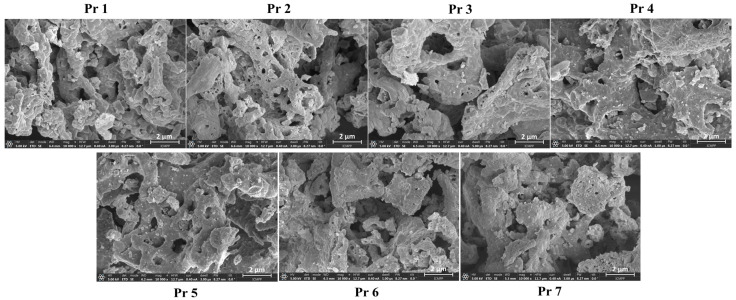
SEM of the samples, scale bar 2 μm.

The SEM images of samples Pr 1–Pr 7 display a spongious appearance with clustered particles. The aspect of the undoped sample is similar to the one of the doped sample (Figure 5).

**Figure 6:**
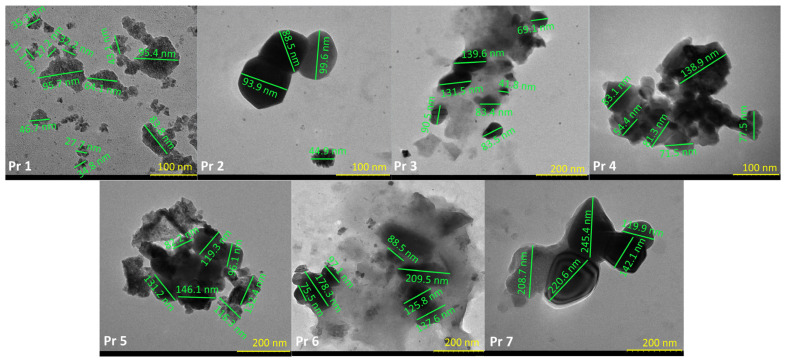
TEM of the samples.

The TEM images present a rough estimate of the particle size range for the obtained doped and undoped cobalt ferrite nanoparticles. For the sample that contains undoped cobalt ferrite, the particle diameters are located between 27 and 64 nm. Some particle conglomerates that reach up to 96 nm are also observable (Figure 6). In the case of doped samples, the range of the particles is within the following intervals: Pr 2—45–100 nm; Pr 3—41–140 nm; Pr 4—54–140 nm; Pr 5—82–146 nm; Pr 6—75–210 nm; Pr 7—119–246 nm. An apparent dopant quantity-dependent diameter increase can also be observed (Figure 6).

### 3.5. Energy Dispersive X-Ray Spectroscopy (EDAX)

**Figure 7:**
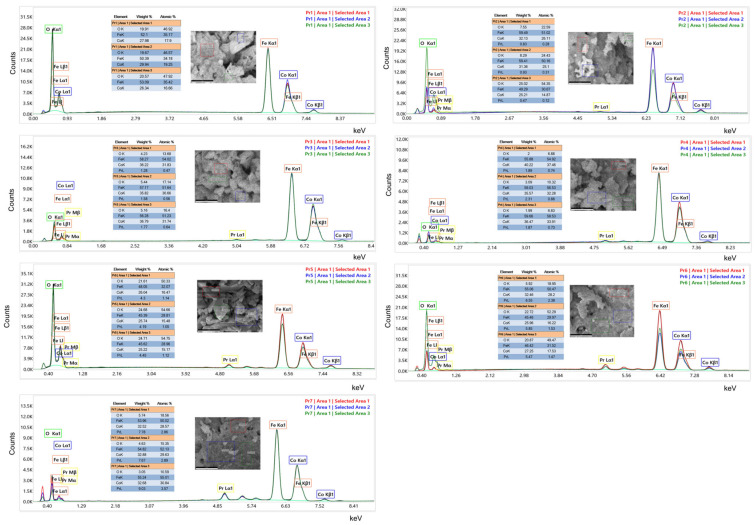
EDAX of the samples.

The peaks identified on the spectra are the ones characteristic of the cobalt ferrite; in all samples, the FeKα is located around 6.4 keV and the CoKα at approximately 6.9 keV. The peak for FeKβ is also present at 7.0 keV and, as can be noticed, is overlapped with the CoKα. Also, the peak for CoKβ, of lower intensity, appears around 7.6 keV. In the domain 0–1 keV, the Kα line for oxygen and the L lines for cobalt and iron are located. Regarding the oxygen content, the difficulty of EDAX to quantify light elements must be stated. In the case of doped samples, the L and M lines for Pr are present, with a visible and distinct peak PrLα at a value of 5–5.2 keV, as well as the M lines overlapped in the 0–1 keV region (Figure 7).

### 3.6. Cell Viability

**Figure 8:**
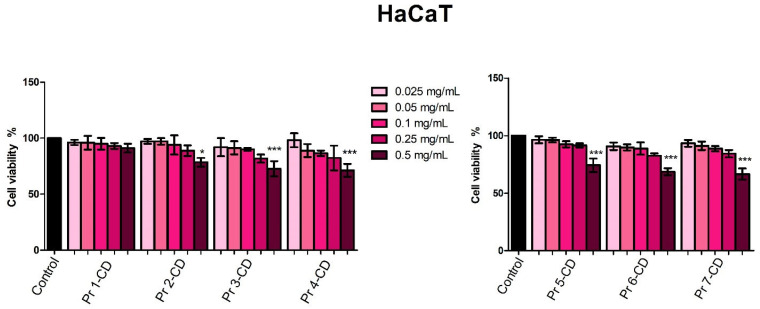
HaCaT cell viability 48 h post-treatment with Pr 1-CD, Pr 2-CD, Pr 3-CD, Pr 4-CD, Pr 5-CD, Pr 6-CD, and Pr 7-CD (0.025, 0.05, 0.1, 0.25, and 0.5 mg/mL). The results are expressed as viability percentages compared to the control group (100%) (* *p* < 0.05, *** *p* < 0.001). The data represent the mean values ± SD of three independent experiments performed in triplicate.

The viability of human keratinocytes HaCaT, human melanoma A375, human breast adenocarcinoma MCF-7, and human colorectal adenocarcinoma HT-29 cells was evaluated 48 h post-treatment with the newly synthesized compounds (0.025, 0.05, 0.1, 0.25, and 0.5 mg/mL) using Alamar blue assay. The incubation of HaCaT cells revealed that Pr 1-CD did not alter cell viability at any of the concentrations tested, whereas Pr 2-CD, Pr 3-CD, Pr 4-CD, Pr 5-CD, Pr 6-CD, and Pr 7-CD altered cell proliferation only when used at 0.5 mg/mL, the highest concentration tested (Figure 8).

**Figure 9:**
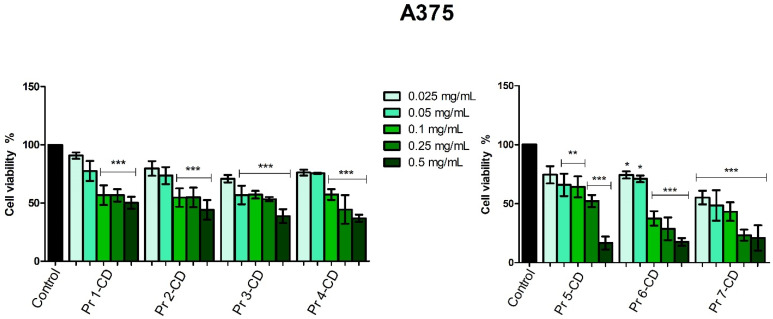
A375 cell viability 48 h post-treatment with Pr 1-CD, Pr 2-CD, Pr 3-CD, Pr 4-CD, Pr 5-CD, Pr 6-CD, and Pr 7-CD (0.025, 0.05, 0.1, 0.25, and 0.5 mg/mL). The results are expressed as viability percentages compared to the control group (100%) (* *p* < 0.05, ** *p* < 0.01, *** *p* < 0.001). The data represent the mean values ± SD of three independent experiments performed in triplicate.

In melanoma cells, Pr 2-CD, Pr 3-CD, Pr 4-CD, Pr 5-CD, Pr 6-CD, and Pr 7-CD decreased cell viability in the most aggressive manner, expressing the lowest IC_50_ values (Table 3). Moreover, the other compound, Pr 1-CD also exerted significant inhibitory effects against A375 cells (Figure 9), particularly in high concentrations, as follows: 56.63% ± 11.91 (0.25 mg/mL), and 50.21% ± 11.29 (0.5 mg/mL).

**Figure 10:**
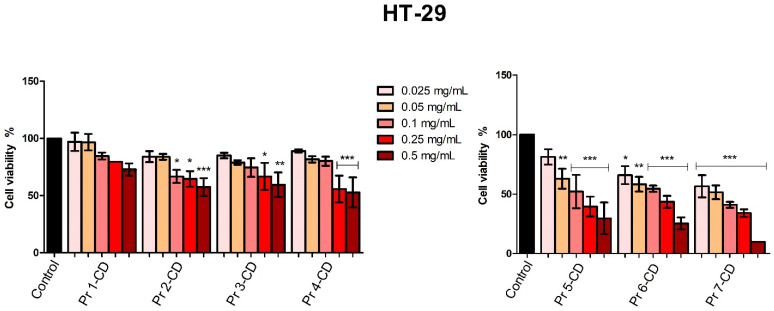
HT-29 cell viability 48 h post-treatment with Pr 1-CD, Pr 2-CD, Pr 3-CD, Pr 4-CD, Pr 5-CD, Pr 6-CD, and Pr 7-CD (0.025, 0.05, 0.1, 0.25, and 0.5 mg/mL). The results are expressed as viability percentages compared to the control group (100%) (* *p* < 0.05, ** *p* < 0.01, *** *p* < 0.001). The data represent the mean values ± SD of three independent experiments performed in triplicate.

When tested against colorectal adenocarcinoma HT-29 cells, Pr 5-CD (IC_50_ 0.13 mg/mL), Pr 6-CD (IC_50_ 0.17 mg/mL), and Pr 7-CD (IC_50_ 0.049 mg/mL) induced the strongest antiproliferative effects, particularly when used in high concentration (Figure 10). Pr 1-CD, Pr 2-CD, Pr 3-CD, and Pr 4-CD also decreased significantly cell viability, as follows: 72.95% ± 10.53 (Pr 1-CD 0.5 mg/mL), 57.45% ± 15.57 (Pr 2-CD 0.5 mg/mL), 59.48% ± 21.43 (Pr 3-CD 0.5 mg/mL) and 52.82% ± 29.23 (Pr 4-CD 0.5 mg/mL).

**Figure 11:**
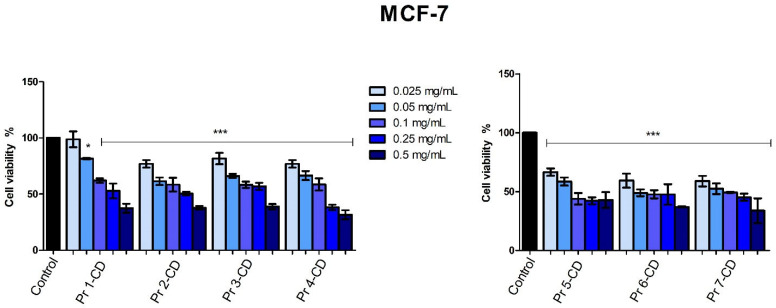
MCF-7 cell viability 48 h post-treatment with Pr 1-CD, Pr 2-CD, Pr 3-CD, Pr 4-CD, Pr 5-CD, Pr 6-CD, and Pr 7-CD (0.025, 0.05, 0.1, 0.25, and 0.5 mg/mL). The results are expressed as viability percentages compared to the control group (100%) (* *p* < 0.05, *** *p* < 0.001). The data represent the mean values ± SD of three independent experiments performed in triplicate.

The stimulation of breast adenocarcinoma MCF-7 cells for 48 h with the tested samples showed that all compounds reduced cell viability in a dose-dependent manner, with Pr 6-CD and Pr 7-CD exerting the strongest anticancer effect as indicated by an IC_50_ value of 0.008 mg/mL. All the newly synthesized compounds decreased MCF-7 cell viability (Figure 11) at an accelerated rate when the highest tested concentrations were applied, as follows: 37.34% ± 8.09 (Pr 1-CD 0.5 mg/mL), 37.7% ± 3.29 (Pr 2-CD 0.5 mg/mL), 38.77% ± 4.72 (Pr 3-CD 0.5 mg/mL), 31.53% ± 7.95 (Pr 4-CD 0.5 mg/mL), 42.97% ± 6.70 (Pr 5-CD 0.5 mg/mL), 37.06% ± 1.87 (Pr 6-CD 0.5 mg/mL), 33.79% ± 10.49 (Pr 7-CD 0.5 mg/mL).

The selectivity index (SI) was calculated for each compound as the ratio between the IC_50_ values obtained in normal HaCaT cells and the IC_50_ value obtained in A375, HT-29, and MCF-7 cancer cell lines (IC_50_ HaCaT/IC_50_ A375, HT-29, MCF-7) [22]. The obtained results show that all tested compounds have an SI higher than 1 (Table 4), thus indicating a desirable selectivity against cancer cells.

## 4. Discussion

In the current study, we aimed to achieve the synthesis of cobalt ferrite nanoparticles doped with Pr in the effort to identify therapeutic alternatives to fight melanoma, colorectal adenocarcinoma, and breast cancer. Although conventional therapies that combine cancer staging with a multitude of strategies (chemotherapy, radiation, surgery) can produce satisfying outcomes for a limited period of time, they also come with severe side effects due to a lack of selectivity and may also induce drug resistance. Additionally, conventional anticancer drugs may exhibit poor pharmacokinetic profiles that may be associated with partial lack of efficacy or toxicity. Such challenges can be overcome by using nanoformulations that provide benefits in terms of bioavailability, stability, and administration [23]. Among the huge number of metallic nanoparticles, cobalt ferrites have gained increased interest due to their high coercivity at room temperature, combined with moderate magnetization [24]. Cobalt ferrites are spinel nanoparticles that have been tested as therapeutic agents, both as drug carriers and hyperthermia agents [25]; however, their intrinsic anticancer properties have been poorly investigated so far. One such study revealed their ability to reduce the viability of MCF7 breast cancer cells in a dose-dependent manner, with stronger effects compared to magnesium-doped cobalt ferrites [26]. Such effects could be triggered by the intrinsic toxicity of cobalt ions that may act as cytotoxic agents, following their release as a result of lysosome degradation of the cobalt ferrite nanoparticle in an acidic environment [26]. The use of cobalt ferrites as anticancer agents is, therefore, worthy of investigation, particularly considering their ability to combine heat and cytotoxic effects into one single platform. Moreover, the introduction of small amounts of rare earth metals into the lattice of cobalt ferrites can tune their magnetic, electronic, and cytotoxic properties, presumably due to the complex interactions between the 3d cation orbitals and 2p oxygen orbitals as a result of metal distribution over the tetragonal and octahedral positions [27]. Based on previous promising results with dysprosium doping [5], we chose to prepare Pr-doped cobalt ferrite nanoparticles whose physicochemical and biological analysis was conducted; their low water solubility introduced the challenge of facilitating their uptake by the aqueous biological medium, which was solved by adding the hydrophilic HPGCD.

A multitude of synthetic approaches have been designed to achieve cobalt ferrite nanoparticles, including sol-gel emulsion or auto-combustion, co-precipitation, gamma-irradiation, and microwave-mediated, hydrothermal, and thermal decomposition [28], displaying various challenges, such as the use of toxic reagents or uncontrollable properties of the resulting nanoparticles. The combustion method used in the current study provides undisputable advantages in terms of feasibility (single-step method, easily applicable and not expensive) but also quality of the final product: high-crystallinity, homogenous ferrites where the intrinsic energy of the components enables the occurrence of metastable phases in one step [29]. Additionally, through controlling the reaction parameters, one can modulate the properties of the final nanoparticles [30]. Also, the sol-gel method offers excellent scalability for biomedical applications due to its adaptability to large-scale synthesis and produces homogeneous nanostructures, making it ideal for drug delivery in clinical settings [19].

The X-ray diffraction analysis revealed the formation of cubic cobalt ferrite and the successful incorporation of Pr in the ferrite structure.

The reaction conditions used for the synthesis of CoFe_2-y_Pr_y_O_4_ led to the formation of a single cubic cobalt ferrite Fd-3m phase for samples Pr 1, Pr 2, and Pr 3 (y = 0; 0.01 and 0.03, respectively) and to the occurrence of a secondary orthorhombic Pbnm phase for samples Pr 4 Pr 5, Pr 6, and Pr 7 (y = 0.05; 0.1; 0.15; 0.2, respectively). The formation of the secondary phase did not produce a visible decrease in the peak intensity in Pr-doped cobalt ferrites, but its occurrence was previously reported in similar studies [31,32].

Our results are in agreement with those reported by Pachpinde et al. [33] who synthesized Pr_x_CoFe_2-x_O_4_ (x = 0–0.1) by the sol-gel auto combustion method using citric acid and nitrates as precursors. They showed that for x ˂ 0.05 Pr was successfully incorporated into the ferrite lattice, while for values above 0.05, a secondary phase of perovskite PrFeO_3_ was found alongside the cubic cobalt ferrite. An explanation for the occurrence of the secondary phase was provided by Nikmanesh et al. [32] who investigated the effect of Pr doping on the cobalt ferrite structure. The occurrence of the secondary phase can be a result of the difference between the ionic radius of iron and the substituting Pr, which limits the dissolution of Pr^3^⁺ in the spinel structure; similar conclusions were drawn in other studies where various lanthanides were doped in ferrite structures [34,35,36].

The lattice parameter determined by Rietveld refinement revealed an increase in the spinel’s cubic system with the increase in Pr^3+^. This can be explained by the presence of Pr^3+^ with a larger atomic radius that led to dilatation of the unit cell and, therefore, of the lattice parameter [33]. The presence of characteristic bands for Pr^3+^ on EDAX, corroborated by the increase in the lattice parameter, confirms once again the successful incorporation of Pr^3+^ into the lattice. Moreover, we observed that increase in the lattice parameter that is associated with a higher Pr content is correlated to a decreased magnetization (Mr, Ms) due to Pr^3+^ replacement of Fe^3+^ from the structure and an increase in the cytotoxic effect; similar findings are presented in the case of Mn_x_Fe_3-x_O_4_ (x = 0, 0.3, 0.5, 0.7) [37].

In the next step, the FTIR spectra were built, revealing a strong absorption band around 570 cm^−1^ in each investigated sample, with a shoulder present around 400 cm^−1^, characteristic of the stretching vibrations attributed to the tetrahedral vibration (metal–oxygen in site A) and the octahedral vibration (metal–oxygen in site B), respectively [38]. We noticed that with the increase in Pr^3+^ as dopant in cobalt ferrite, a shift in the absorption band from 572 cm^−1^ to 567 cm^−1^ occurred. This band is associated with the M-O stretching vibrations that take place in the tetrahedral site of the spinel lattice. This shift could indicate a weakening of the M-O bond located at this site. The incorporation of Pr^3+^ in the spinel structure can lead to lattice distortion and cation redistribution between sites [32]. Pr^3+^ has a larger ionic radius (1.13 Å) compared to Fe^3+^ ionic radius (0.67 Å) and prefers to occupy the octahedral sites. After this substitution, the Fe^3+^ ions that are pulled out from the octahedral site could eventually migrate to a tetrahedral site, leading to a change in cation distribution and an alteration of the M-O bond (length, strength) from the tetrahedral site [39,40]. In a series of trivalent ions of rare earth metals (Gd, Sm, Ho, Er, Yb, Y) used as dopants for cobalt ferrite, Jing et al. [35] emphasized that the absorption band around 570 cm^−1^ shifts to higher wavenumbers for samarium, holmium, erbium, ytterbium and yttrium, while for gadolinium, the shift occurs toward lower wavenumbers. Moreover, they assigned the 570 cm^−1^ band to the tetrahedral stretching vibration between M-O and the second band to the octahedral stretching vibration between metal and oxygen. Additionally, the samples that contain Pr exhibited a shift toward lower wavenumbers, e.g., 567 cm^−1^ in Pr 7. A similar observation was previously reported in Pr-doped cobalt ferrite [33] as well as in dysprosium-doped cobalt ferrite [5], where the wavenumber decreased with the increase in the rare earth metal substitution. The replacement of iron by Pr, with a larger ionic radius, resulted in an increased unit cell dimensions and altered the iron–oxygen vibrations, thus leading to a change in band positions. The peak intensity also changed with an increased Pr^3+^ substitution; the change in the frequency of the second stretching band points to the preferred orientation of Pr^3+^ ions toward octahedral sites. Also, the slight decrease in the second band frequency may be attributed to the presence of the secondary phase in samples with a higher Pr^3+^ substitution level [33].

The VSM analysis revealed that the presence of Pr in the ferrite lattice reduced the Ms and Mr values, particularly in samples where cyclodextrin was used as coating material; such intense magnetization reduction can be attributed to the large amount of cyclodextrin that lacks magnetic behavior and is able to shield the intrinsic magnetic properties of the included ferrite. In the case of Pr-doped cobalt ferrite (sample Pr 2–Pr 7), Pr^3+^ with a larger atomic radius substitutes from the spinel lattice Fe^3+^ with a smaller atomic radius. As mentioned before, this significant difference in the ionic radius of the two elements can lead to the formation of defects, cation redistribution, and lattice distortion in the ferrite structure. We observed that the cell parameter increased with the content of Pr^3+^ due to its larger ionic radius, with similar observations made by Nikmanesh et al. [32]. Pr^3+^ tends to preferentially occupy the octahedral sites from the lattice, and this could modify the cation distribution throughout the tetrahedral and octahedral sites, with Fe^3+^ migration from octahedral to tetrahedral positions, thereby impacting the structure and properties [33,41]. When Pr^3+^ replaces magnetic ions such as Fe^3+^ or Co^2+^ in the system, the magnetic properties are impacted by the resulting decrease in magnetic moment [42]. Furthermore, these substitutions can impact the super-exchange interactions between A and B sites, such as Fe^3+^(A)-O^2−^-Fe^3+^(B), Fe^3+^(A)-O^2-^-Co^2+^(B), which dictates the ferrimagnetism of the compound [42,43,44,45]. The presence of orthoferrite PrFeO_3_ as the secondary phase contributes to the decrease in the total magnetization and can hinder the interactions between elements and sites, which usually take place in the lattice [41]. Furthermore, the lattice strain resulting from the Pr^3+^ substitution can alter the magnetic interaction and domain structure, leading to nonlinear variation in Ms and Mr [32]. Similar data were reported for samples of CoFe_2−x_Pr_x_O_4_ (x = 0, 0.02, 0.04, 0.06) [32] where the Ms values decreased with the increase in Pr content; as an example, for x = 0, Ms was around 80 emu/g and reached 60 emu/g for x = 0.06. In our study, the Ms value for x = 0 was around 73 emu/g and dropped to 66 emu/g for x = 0.05. Vani et al. [45] studied the effect of terbium ions (Tb^3+^) on the cobalt ferrite structure and properties; they reached a similar conclusion in terms of magnetic properties, reporting that the value of Ms decreases with rising terbium concentration, presumably due to the substitution of the highly magnetic iron with the less magnetic terbium. Such conclusions were supported by other studies on rare earth metal-doped cobalt ferrites, where the values of both Ms and Mr could be clearly correlated with the content of the doping material [5,40,46].

The morphology of the samples is illustrated in the SEM images; a sponge-like, foam-like cavernous structure is typical of cobalt ferrite obtained by combustion synthesis. Similar morphological features were previously reported by Lin et al. [47] for gadolinium-doped cobalt ferrite and by Abbas et al. [48] for aluminum-doped cobalt ferrite, both synthesized by the sol-gel auto-combustion method. Although the SEM images of undoped and doped samples appear similar, the TEM images clearly reveal that the presence of the dopant influences the particle size and shape. Specifically, TEM analysis revealed an increase in particle size with higher Pr doping levels, which is correlated with lower IC_50_ values and, hence, improved cytotoxicity in cancer cell lines. The observed increase in cytotoxicity is not directly attributed to the larger particle size but rather to the higher Pr content. Smaller particle sizes are generally associated with enhanced anticancer effects; the threshold vesicle size for tumor extravasation was revealed to be ∼400 nm, and particles with diameters below 200 nm have demonstrated a more pronounced cytotoxic effect [49]. The increase in particle size was accompanied by an increase in dopant levels, ranging from 24 to 64 nm for the pure sample, increasing to 120–245 nm for sample Pr 7, which has the highest amount of the dopant. Similar findings were presented by Yadav et al. [50] in the study regarding the synthesis of CoFe_2−x_Pr_x_O_4_ (x = 0.0, 0.025, 0.05, 0.075, 0.1) using starch-assisted sol–gel auto-combustion. Starting with a range of 5-10 nm for the undoped sample, the particle dimensions doubled in size for a content of Pr of 0.075. Manikandan et al. [51] used a chemical oxidation method to synthesize pure and doped cobalt ferrite, using Pr in different ratios between 0 and 5%. The FESEM results indicated spherical particles with a range between 30 and 160 nm in the case of a pure sample. When Pr was added to the sample, and the doping percentage increased up to 5%, an increase in particle size ranging from 70 to 200 nm was noted. Furthermore, in the case of zinc–cobalt ferrite [41], Pr-doping resulted in both an increased particle size and altered particle morphology. Comparable findings were also reported in the case of gadolinium-doped cobalt ferrite CoFe_2−x_Gd_x_O_4_ (x = 0–0.30) [52]. The TEM images also indicate a tendency toward particle agglomeration or even superposition. Variations in particle aggregation, shape, and dimensions can impact cellular uptake and biological interactions. In our case, these variations might explain the fact that some IC_50_ values of the Pr-doped cobalt ferrite–cyclodextrin conjugates do not follow a strictly linear trend with increasing Pr content (Table 2).

The biological evaluation of the newly synthesized Pr-doped cobalt ferrite nanoparticles as cyclodextrin inclusion complexes was conducted both on normal human keratinocytes cell line and three cancer cell lines, namely, human melanoma, human breast adenocarcinoma, and human colorectal adenocarcinoma cell lines. This strategy provides insights into the efficacy of the tested samples as anticancer agents while also assessing their selectivity. Reference to previously reported data is challenging since, to the best of our knowledge, this is the first biological evaluation of Pr-doped CoFe_2_O_4_ nanoparticles. The biological assessment in HaCaT normal human keratinocytes revealed that the tested compounds exhibited low cytotoxic, dose-dependent activity; thus, an antiproliferative activity was only displayed when the highest concentration (0.5 mg/mL) of the tested compounds was applied. Moreover, along with the high IC_50_ values (>0.5 mg/mL) obtained and a SI > 1, the results indicate high biocompatibility of the tested compounds and their potential selective anticancer activity, which stands as an important parameter in the development of new anticancer agents. The high biocompatibility of our compounds when tested in HaCat cells is also in line with previous data on cobalt ferrite nanoparticles that reported low cytotoxicity even at concentrations of 2 mg/mL [53], with significant reductions in cell viability only at concentrations as high as 4 mg/mL, combined with 72 and 96 h incubation time [53].

The evaluation of Pr-doped cobalt ferrite nanoparticles’ cytotoxic effects in A375 human melanoma cells revealed a dose-dependent antiproliferative activity, particularly after treatment with the highest concentration of each compound (0.5 mg/mL). The intensity of the cell viability inhibitory activity correlates with the amount of Pr in the tested cobalt ferrite nanoparticles; cytotoxic activity increased exponentially with the amount of Pr. The role of Pr in exerting the inhibitory effect is obvious when comparing the doped cobalt ferrites to the undoped ones, in all tested concentrations (e.g., Pr 1-CD vs. Pr 2-CD). To the best of our knowledge, this is the first assessment of Pr-doped cobalt ferrites as anticancer agents; however, we previously conducted similar studies on Dy-doped CoFe_2_O_4_ nanoparticles that revealed a dose-dependent cytotoxic activity against the A375 cell line which was also correlated to the amount of dysprosium used as substitution for the iron ions [5]. Cobalt ferrite nanoparticles were also previously investigated for magnetic hyperthermia and light-based treatments in the eradication of cancer stem cells in the A375 cell line, displaying effective cytotoxicity with IC_50_ values of 0.025 mg/mL [54].

The antiproliferative activity of the tested compounds was confirmed in the HT-29 colorectal adenocarcinoma cell line. When tested in the highest concentration, all compounds displayed a strong dose-dependent cytotoxic activity. Compounds Pr 5-CD and notably Pr 6-CD exhibited exponential cytotoxicity in correlation to an increased Pr substitution, with cell viability values of 29.54% and 25.28%, respectively. Pr 7-CD exhibited the strongest antiproliferative activity in all tested concentrations, decreasing HT-29 cell viability to 9.28% when tested in the highest concentration, presumably due to its high amount of Pr. As we stated previously, we were not able to find another attempt to assess the anticancer activity of Pr-doped cobalt ferrite nanoparticles; however, in a similar study, Pr-substituted Ni-Co nano-spinel ferrites exhibited efficient cell viability inhibition in HCT-116 colorectal carcinoma cells, displaying an average inhibitory activity of 50% [17]. Given that the HCT116 cell line is considered highly aggressive by comparison with the HT29 cell line, which shows an intermediate differentiating potential, we may state that our Pr-doped nanoparticles exhibit similar inhibitory activity to the Pr-doped Ni-Co ferrites. However, in our case, the presence of fewer elements in the metallic nanoparticles structure enables simpler synthesis and reduces the number of experimental variables. Additionally, Nd^3+^ and Ce^3+^ co-substituted Co ferrite nanoparticles tested on the same colorectal carcinoma cell line showed a significantly reduced cell viability through a dose-dependent apoptotic mechanism [55].

When tested on the MCF-7 breast adenocarcinoma cell line, all compounds displayed dose-dependent antiproliferative activity, with IC_50_ values ranging from 0.08 to 0.32 (Table 2). The cytotoxic activity in MCF-7 cells is more evident when compared to the results on the HaCaT cell line, where an IC_50_ value >0.5 was obtained for all compounds, thus emphasizing the selectivity of the tested compounds against MCF-7 cells. An overview of cell viability inhibition in all tested cancer cell lines, with respect to their IC_50_ values (Table 2), revealed that the MCF-7 cell line was the most responsive to the antiproliferative effect exerted by the Pr-doped CoFe_2_O_4_ nanoparticles. When compared to our previous results on Dy-doped CoFe_2_O_4_ nanoparticles, the Pr-doped CoFe_2_O_4_ nanoparticles displayed higher cytotoxicity, reducing MCF-7 cell viability to less than 50% when using half the concentration [5]. Pr-metal nanorods coated with poly-CD polymer were synthesized as a carrier for 5-fluorouracil and investigated in terms of their anticancer potential against the MCF-7 cell line; they were revealed to be a suitable vehicle because they provided sustained release for the incorporated drug [18]. Also, cobalt ferrites were investigated as drug carriers in MCF-7 cells; one study involved the use of cobalt ferrites for the delivery of docetaxel, where an intrinsic cytotoxicity of the bare nanoparticles was reported [56]. In addition, cobalt ferrite nanoparticles demonstrated a mild antiproliferative activity against the MCF-7 cancer cell line, promoting a mean viability of 74–85% in all tested concentrations [57]. It is important to note, however, that the enhanced inhibitory activity of Pr-doped cobalt ferrites could be previewed if considering the promising results reported by Andiappan et al. when investigating the anticancer effects of Pr-doped Schiff bases in several cancer cells; they revealed that the metal ions (Pr^3+^) exhibited strong affinity for the cell walls, thus leading to DNA fragmentation and cell cycle arrest [58]. Additionally, a polymeric quadrivalent Pr complex was tested in seven cancer cell lines, and although weaker than conventional anticancer agents, it induced cytotoxic effects in all types of cancer cells but particularly in A375 melanoma cells [59]. Exposure of cancer cells to low concentrations of a small coordinated complex of Pr and pyrithione significantly diminishes cell survival regardless of drug-resistant phenotype by affecting the cell waste-clearing mechanisms and by disrupting mitochondrial metabolism [60]. Collectively, our data corroborate with previously published results in terms of the anticancer effects of both Pr and cobalt ferrites.

The presence of a secondary orthorhombic PrFeO_3_ phase in higher-doped samples (Pr 4–Pr 7), as identified via XRD, contributes to the decrease in the total magnetization and might be a critical factor influencing anticancer performance. The observed magnetic dilution effect, enhanced by cyclodextrin surface modification, contributes to a soft magnetic behavior that may be advantageous for biomedical applications [61]. Interestingly, in our case, despite a reduction in magnetization and the presence of this secondary phase, Pr 6-CD and Pr 7-CD exhibited the most potent antiproliferative effects across all cancer cell lines tested. The exact contribution of magnetic dilution effect and of PrFeO_3_ secondary phase to the observed anticancer effects cannot be conclusively established based on the current data, and further investigation is warranted.

The cyclodextrin coating presumably acted as a stabilizer for the metallic nanoparticles [62]. Additionally, the presence of the cyclodextrin coating increased the aqueous solubility of the nanoparticles, which presumably induced a higher ability to concentrate in the biological intracellular environment, thus inducing higher cellular uptake and stronger cytotoxic activity. The cyclodextrin layer also exhibits functional hydroxyl groups that can be derivatized in order to attach various ligands; its cavities can accommodate various anticancer drugs that can thus be carried and delivered to the tumor site [63].

In terms of the underlying mechanism of action, there are several studies that report apoptosis, induced through different pathways, as being responsible for the cytotoxic properties of various types of doped cobalt ferrites [55,64]. Horev-Azaria et al. [65] reported that Co-Fe NPs can induce oxidative stress by increasing ROS generation in various cancer cell lines. In MCF-7 breast cancer cells, nickel-doped Co ferrite nanoparticles have been reported to induce apoptosis by increasing ROS generation, downregulating Bcl-2 expression, and upregulating p53, Bax, as well as cleaved caspase-3, -8, and -9 protein levels [66]. In a previous study, our group reported that Dy-doped Co ferrites had cytotoxic effects and induced apoptosis in A375 and MCF-7 cancer cells [5]. Specifically, Dy-doped Co ferrites decreased the expression of cyclin D1, increased caspase-9 level, and upregulated the p53 protein; these effects became more pronounced with increasing Dy substitution, with the most significant changes observed at the highest levels of iron ion replacement by Dy in the doped ferrites [5]. All data considered, the newly synthesized CoFe_2−γ_Pr_γ_O_4_ nanoparticles demonstrate promising selective anticancer activity against A375, MCF-7, and HT-29 cell lines, thus emphasizing the potential of Pr-doped nanoparticles to act as an effective alternative to conventional treatments. However, further mechanistic studies are warranted to elucidate the precise molecular pathways involved. This need is underscored by the related literature reporting apoptosis induction, ROS generation, and modulation of apoptotic proteins in similar rare-earth-doped ferrite systems, which could potentially support and guide future investigations into the observed cytotoxic effects.

## 5. Conclusions

The present study successfully presents the synthesis of Pr-substituted cobalt ferrites (CoFe_2−y_Pr_y_O_4_, y = 0–0.2) and the characterization of their structural, morphological, magnetic, and biological properties. X-ray diffraction confirmed the formation of single-phase cubic cobalt ferrite at low Pr levels, with a secondary phase appearing at higher doping. FTIR showed characteristic metal–oxygen bonds shifting with increased Pr content. Magnetic measurements revealed decreasing magnetization with doping and cyclodextrin coating, while TEM and SEM analyses indicated increasing particle size and porous morphology. The in vitro studies revealed a selective cytotoxic effect against melanoma, breast, and colorectal cancer cells, with minimal effects on normal human keratinocytes. Noteworthy, the samples Pr 6-CD and Pr 7-CD exhibited the most antiproliferative activity, with the lowest IC_50_ values. This enhanced activity may be associated with Pr substitution level, particle size, morphology, and surface properties, all of which support the need for further investigations to fully elucidate their underlying molecular mechanisms and to validate their clinical relevance. Overall, the study underscores the potential of Pr-doped cobalt ferrite nanoparticles, particularly those surface-modified with cyclodextrin, as multifunctional platforms for targeted biomedical applications.

## Figures and Tables

**Table 1:** The sample composition.

Sample	PrCl_3_·xH_2_O (moles)	Fe(NO_3_)_3_·9H_2_O (moles)
Pr 1	-	0.0400
Pr 2	0.0002	0.0398
Pr 3	0.0006	0.0394
Pr 4	0.0010	0.0390
Pr 5	0.0020	0.0380
Pr 6	0.0030	0.0370
Pr 7	0.0040	0.0360

**Table 2:** The calculated lattice parameter *a* and Scherrer crystallite size.

Sample	Lattice Parameter *a* (Å)	Crystallite Size (nm)
Pr 1	8.3749 ± 0.0003	55.95 ± 0.5
Pr 2	8.3773 ± 0.0004	43.45 ± 0.4
Pr 3	8.3788 ± 0.0004	49.46 ± 0.5
Pr 4	8.3809 ± 0.0005	48.58 ± 0.6
Pr 5	8.3851 ± 0.0004	60.38 ± 0.9
Pr 6	8.3873 ± 0.0004	65.59 ± 0.9
Pr 7	8.3879 ± 0.0005	56.20 ± 0.6

**Table 3:** The calculated IC50 values (mg/mL) of Pr 1-CD, Pr 2-CD, Pr 3-CD, Pr 4-CD, Pr 5-CD, Pr 6-CD, and Pr 7-CD on HaCaT, A375, HT-29, and MCF-7 cell lines 48 h post-stimulation.

Compound	HaCaT	A375	HT-29	MCF-7
Pr 1-CD	>0.5	>0.5	>0.5	0.25
Pr 2-CD	>0.5	0.28	>0.5	0.19
Pr 3-CD	>0.5	0.20	>0.5	0.32
Pr 4-CD	>0.5	0.18	>0.5	0.14
Pr 5-CD	>0.5	0.22	0.13	0.12
Pr 6-CD	>0.5	0.08	0.17	0.08
Pr 7-CD	>0.5	0.05	0.049	0.08

**Table 4:** The calculated SI of Pr 1-CD, Pr 2-CD, Pr 3-CD, Pr 4-CD, Pr 5-CD, Pr 6-CD, and Pr 7-CD against A375, HT-29, and MCF-7 cell lines.

Compound	A375	HT-29	MCF-7
Pr 1-CD	1.83	1.49	4.12
Pr 2-CD	3.32	1.60	4.89
Pr 3-CD	3.90	1.13	2.43
Pr 4-CD	4.61	1.36	5.92
Pr 5-CD	4.22	7.15	7.75
Pr 6-CD	11.87	5.58	11.87
Pr 7-CD	16.6	21.22	13

## Data Availability

The original contributions presented in the study are included in the article; further inquiries can be directed to the corresponding author.
